# Supplementary figures and images for: Persistence of chikungunya ECSA genotype and local outbreak in an upper medium class neighborhood in Northeast Brazil
Source: PLoS One. 2020 Jan 8;15(1):e0226098. doi: 10.1371/journal.pone.0226098 (PMC6948741; doi:10.1371/journal.pone.0226098)

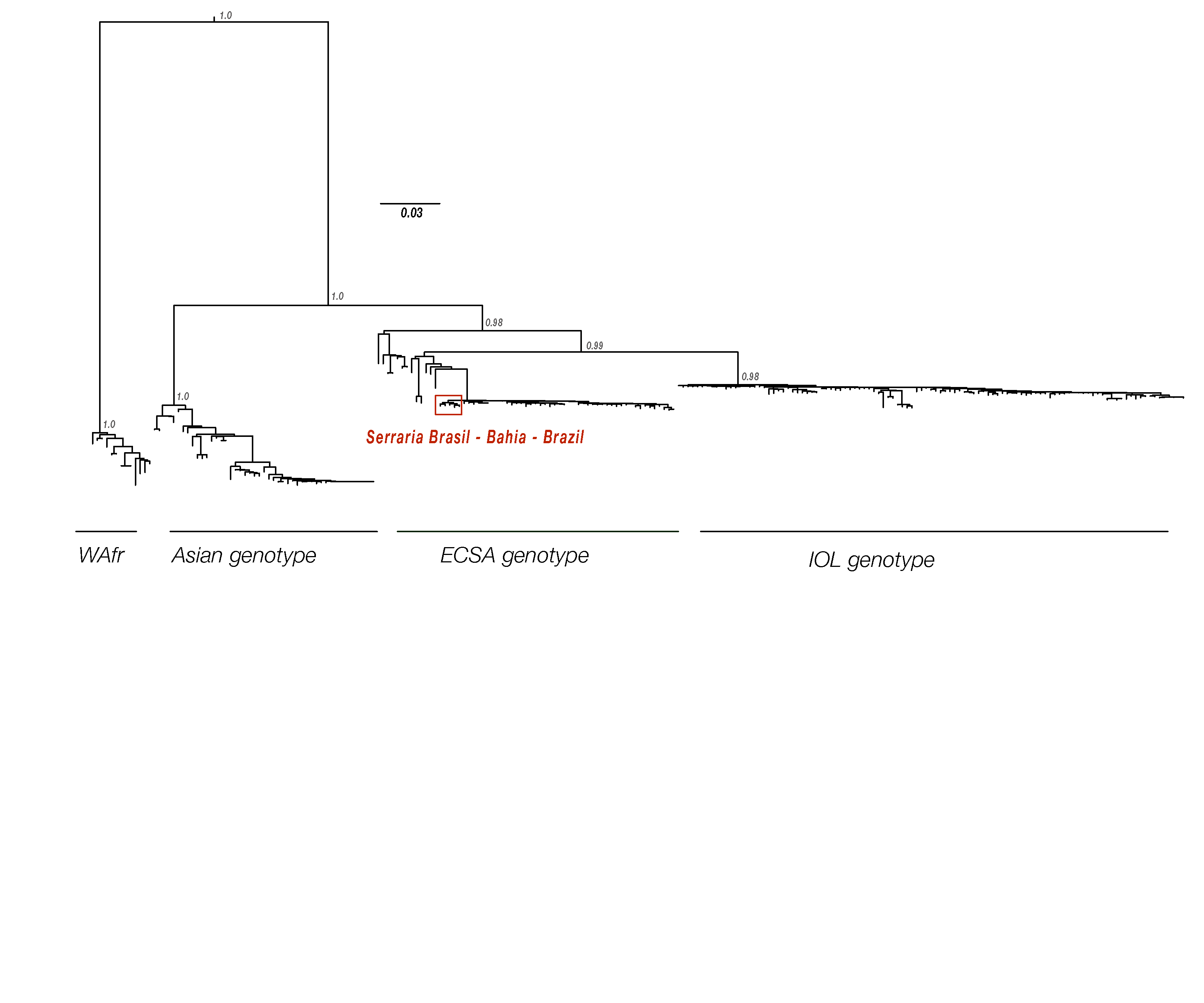

Supplement: S1 Fig — (TIF) [file pone.0226098.s001.tif]

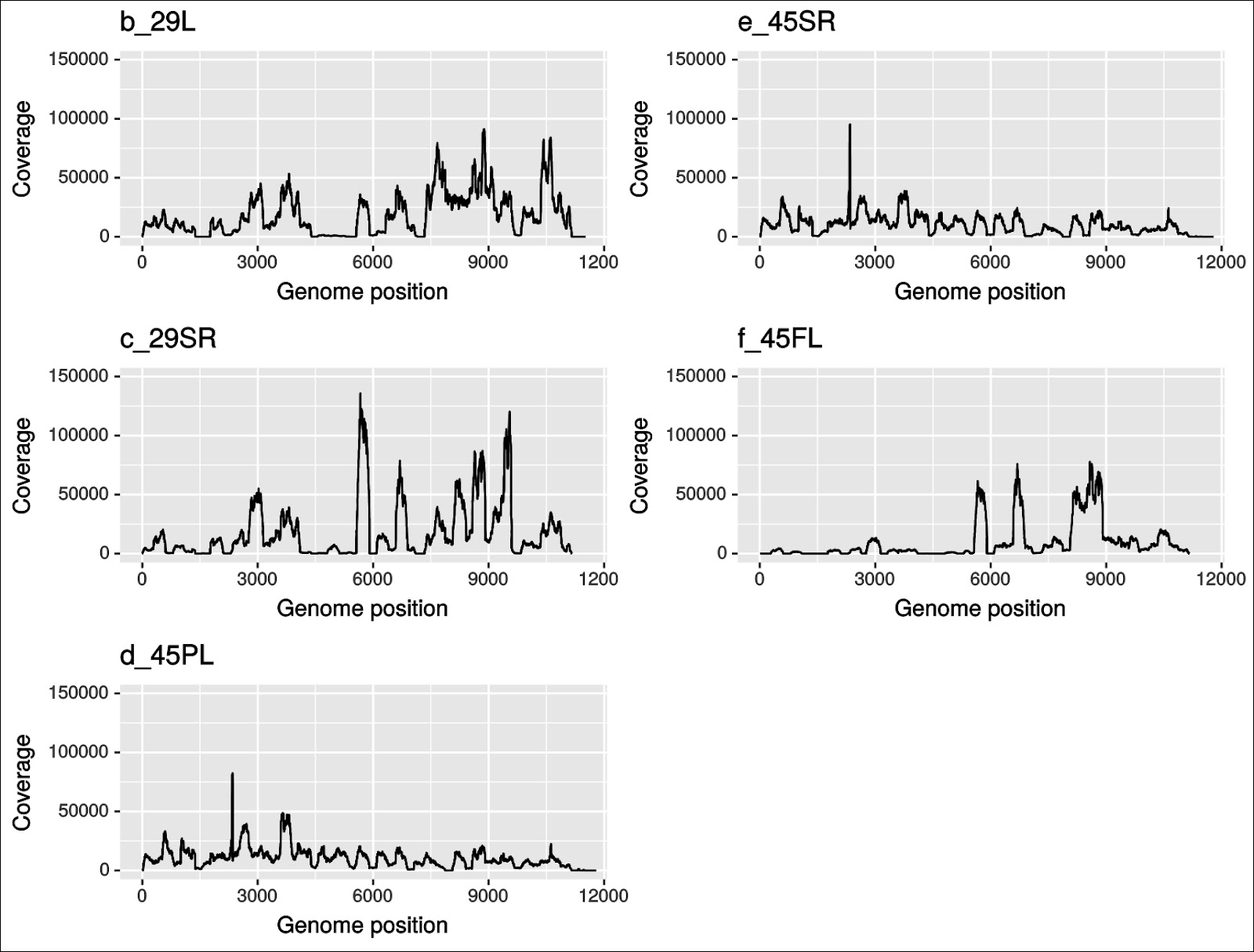

Supplement: S2 Fig — (TIFF) [file pone.0226098.s002.tiff]

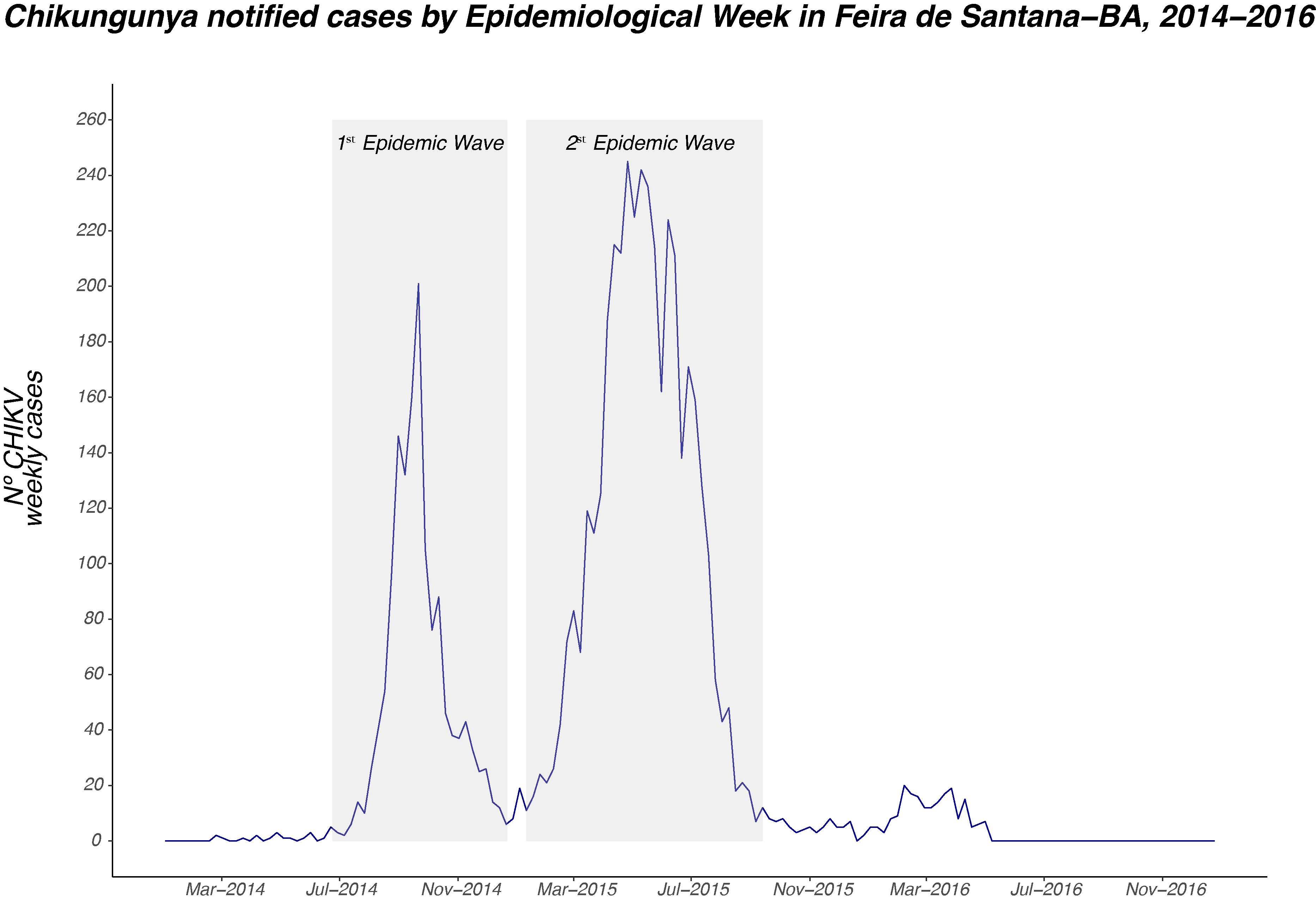

Supplement: S3 Fig — (TIF) [file pone.0226098.s003.tif]
