# Supplementary material for: Persistence of chikungunya ECSA genotype and local outbreak in an upper medium class neighborhood in Northeast Brazil
Source: PLoS One. 2020 Jan 8;15(1):e0226098. doi: 10.1371/journal.pone.0226098 (PMC6948741; doi:10.1371/journal.pone.0226098)
Supplement: S1 Table — (PDF) [file pone.0226098.s004.pdf]

**Table S1. Clinical data of cases included in the study**.

| Subject | Sample | Symptoms | CHIKV RT-PCR | CHIKV IgG Serology | CHIKV IgM Serology |
|---|---|---|---|---|---|
| **1** | plasma, saliva, serum and urine | backache, conjunctivitis, exanthema, fever, headache, muscular pain, nausea, retro-orbital pain | NEG | NEG | NEG |
| **2** | plasma, saliva, serum and urine | exanthema, fever, headache, intense arthralgia, muscular pain, nausea, vomiting | NEG | NEG | POS |
| **3** | saliva, serum and urine | backache, exanthema, headache, intense arthralgia, muscular pain, vomiting | NEG | NEG | POS |
| **4** | plasma, saliva, serum and urine | no data available | NEG | not tested | not tested |
| **5** | plasma, saliva, serum and urine | conjunctivitis, exanthema, fever, intense arthralgia, muscular pain, nausea, retro-orbital pain | NEG | NEG | POS |
| **6** | plasma, saliva, serum and urine | conjunctivitis, exanthema, fever, headache, intense arthralgia, muscular pain, nausea,retro-orbital pain, vominting | NEG | POS | POS |
| **7** | saliva, serum and urine | exanthema, fever, headache, intense arthralgia, muscular pain | NEG | NEG | POS |
| **8** | plasma, saliva, serum and urine | no data available | NEG | not tested | not tested |
| **9** | plasma, saliva, serum and urine | headache, fever, muscular pain, exanthema, vomiting, nausea, backache, conjunctivitis, intense arthralgia | NEG | NEG | POS |
| **10** | saliva, serum and urine | headache, exanthema, nausea, muscular pain, backache, intense arthralgia, retro-orbital pain | NEG | NEG | POS |
| **11** | saliva, serum and urine | headache, fever, muscular pain, backache, conjunctivitis, arthritis, intense arthralgia, retro-orbital pain | NEG | not tested | not tested |
| **12** | saliva, serum and urine | headache, fever, exanthema, muscular pain, backache, intense arthralgia, retro-orbital pain | NEG | NEG | IND |
| **13** | plasma, saliva, serum and urine | headache, fever, exanthema, vomiting, nausea, intense arthralgia | NEG | POS | POS |
| **14** | plasma, saliva, serum and urine | headache, fever, muscular pain, vomiting, nausea, backache, intense arthralgia | NEG | NEG | NEG |
| **15** | plasma | headache, fever, muscular pain, vomiting, nausea, backache, intense arthralgia | NEG | not tested | not tested |
| **16** | plasma and serum | headache, fever, muscular pain, vomiting, nausea, backache, intense arthralgia | NEG | NEG | POS |
| **17** | plasma, saliva, serum and urine | headache, fever, muscular pain, exanthema, conjunctivitis, backache, intense arthralgia | POS plasma; POS serum | NEG | POS |
| **18** | plasma, and serum | fever, headache, exanthema, nausea, intense arthralgia, retro-orbital pain | NEG | NEG | POS |

| | | | | | |
|---|---|---|---|---|---|
| **19** | plasma, saliva, serum and urine | fever, muscular pain, headache, exanthema, vomiting, nausea, intense arthralgia | NEG | NEG | POS |
| **20** | plasma, and serum | fever, muscular pain, headache, backache, conjunctivitis, intense arthralgia | NEG | NEG | POS |
| **21** | plasma, saliva, serum and urine | no data available | NEG | not tested | not tested |
| **22** | plasma, and serum | fever, muscular pain, headache, exanthema, nausea, backache, conjuctivitis, intense arthralgia | NEG | NEG | POS |
| **23** | plasma, saliva, and urine | no data available | NEG | not tested | not tested |
| **24** | plasma, saliva, serum and urine | fever, intense arthralgia, headache, exanthema, muscular pain | POS plasma; POS serum; POS saliva | NEG | POS |
| **25** | plasma, soro | fever, muscular pain, headache, exanthema, vomiting, náusea, backache, conjunctivitis, intense arthralgia | NEG | NEG | POS |
| **26** | plasma, saliva, serum and urine | fever, muscular pain, headache, exanthema, backache, conjunctivitis, intense arthralgia, retro-orbital pain | NEG | IND | POS |
| **28** | plasma, saliva, serum and urine | no data available | NEG | not tested | not tested |
