# Supplementary material for: Persistence of chikungunya ECSA genotype and local outbreak in an upper medium class neighborhood in Northeast Brazil
Source: PLoS One. 2020 Jan 8;15(1):e0226098. doi: 10.1371/journal.pone.0226098 (PMC6948741; doi:10.1371/journal.pone.0226098)
Supplement: Attachment — Submitted filename: Response to Reviewers.docx [file pone.0226098.s005.docx]

**Response to reviewers**

PONE-D-19-29145

Persistence of Chikungunya ECSA genotype and local outbreak in an upper medium class neighborhood in Northeast, Brazil.

In this document, we have addressed each review comment separately. We are glad that all editorial and review comments were acknowledged of significant value to our manuscript. We have considered all comments from the reviewers and the editorial board and we believe that the resulting manuscript conforms better with the journal’s guidelines, and its main messages are now clearer.

**Journal requirements**

*Please remove any funding-related text from the manuscript and let us know how you would like to update your Funding Statement.*

Answer: Thank you for this comment, we removed funding-related text from the manuscript (lines 380-385) and we will update our Funding Statement on the online submission form.

**Reviewer #1**

***Please revise carefully the English used.***

Answer: We agree with the reviewer and we have carefully revised the English used. We’ve made changes, specially converting British English to American English in order to meet journal requirements. This include a small change on “neighbourhood” word in the title which now is *"Persistence of Chikungunya ECSA genotype and local outbreak in an upper medium class neighborhood in Northeast, Brazil”*

***What was the considerations for using 2 x 75 bp NGS reagent? Why not using larger read length such as 2 x 150 bp? Longer reads may decrease gaps.***

Answer: We agree with the reviewer about having a lower number of gaps when using longer reads, however it is most of the time true for de novo assembly. We used 2 x 75 just because that was the flowcell we had at the time of sequencing. Moreover, since we reference assembled a compact viral genome with no repetitive regions the small reads does not affected the assembly considerably. It can be observed on the high coverage breadth we obtained in our genomes. Lastly, the gaps were restricted to the 5’ and 3’ UTR regions that are more difficult to amplify and consequently to assembly, showing that the existing gaps was not due to the assembly problems because of the use of 75bp reads.

***Figure 1. Is the zoom representing the city of Feira de Santana or the Serraria Brasil neighborhood? Please state this and add the hype in the map.***

Answer: We thank the reviewer for this comment.

In Figure 1 we show Feira de Santana municipality and the highways network that converge from all Brazilian regions in that place. We believe this is of particular importance for the dissemination of infectious diseases within the country, as the intense movement of people there, may contribute to virus dispersion to other locations. We added information about location on the map when it shows Brazil and the zoom that is showing Feira de Santana, highlighting the road junction.

***In the method section, could the authors add more information regarding the Library prep sequencing for Illumina, this is really fundamental in the field. Did the author used barcode? Have been this barcode pooled in an equimolar fashion after the amplification?***

Answer: We added more information in the material and methods section lines 136-149. Yes, we used barcodes specific to each sample which allowed us to separate the specific reads after sequencing.

**Reviewer #2**

***The Authors state, in lines 347-349 that these data improve intervention strategies. How? Please provide strong evidence.***

Answer: Thank you for your comment. We added discussion about how genomic surveillance can improve knowledge about mosquito-borne viruses and public health strategies (lines 372 – 390).
